# Animal Transmission of SARS-CoV-2 and the Welfare of Animals during the COVID-19 Pandemic

**DOI:** 10.3390/ani11072044

**Published:** 2021-07-08

**Authors:** Kimberly Ekstrand, Amanda J. Flanagan, Ilyan E. Lin, Brendon Vejseli, Allicyn Cole, Anna P. Lally, Robert L. Morris, Kathleen N. Morgan

**Affiliations:** 1 Department of Philosophy, Wheaton College, Norton, MA 02766, USA; ekstrand_kimberly@wheatoncollege.edu; 2 College of Veterinary Medicine, Cornell University, Ithaca, NY 14850, USA; af649@cornell.edu; 3 Department of Biology, Wheaton College, Norton, MA 02766, USA; lin_ilyan@wheatoncollege.edu (I.E.L.); vejseli_brendon@wheatoncollege.edu (B.V.); rmorris@wheatoncollege.edu (R.L.M.); 4 Program in Neuroscience, Wheaton College, Norton, MA 02766, USA; cole_allicyn@wheatoncollege.edu (A.C.); lally_anna@wheatoncollege.edu (A.P.L.)

**Keywords:** COVID-19, SARS-CoV-2, animal transmission, animal model, animal welfare

## Abstract

**Simple Summary:**

The accelerated pace of research into Severe Acute Respiratory Syndrome Coronavirus 2 (SARS-CoV-2) necessitates periodic summaries of current research. The present paper reviews virus susceptibilities in species commonly in contact with humans and predictors of susceptibility. With few exceptions, species selected for review were those in contact with humans through the entertainment, pet, or agricultural trades, and for whom report—either anecdotal or published—exist regarding the SARS-CoV-2 virus and/or the resulting disease state COVID-19. The possibility of humans transmitting SARS-CoV-2 to animals and fear of animals transmitting the virus to humans endangers animal wellbeing; use of animals as research models also has welfare implications. As the search for appropriate animal models for SARS-CoV-2 continues, it is important to determine which species are most appropriate, so that the “three R’s” of animal research (replacement, reduction, and refinement) may be put into practice. The present review suggests that ferrets, golden Syrian hamsters, and some Old World nonhuman primates may be the best animal models for COVID-19-related research, as these species display the greatest similarity in factors underlying viral infection, as well as clinical symptoms upon viral exposure most similar to those seen in humans.

**Abstract:**

The accelerated pace of research into Severe Acute Respiratory Syndrome Coronavirus 2 (SARS-CoV-2) necessitates periodic summaries of current research. The present paper reviews virus susceptibilities in species with frequent human contact, and factors that are best predictors of virus susceptibility. Species reviewed were those in contact with humans through entertainment, pet, or agricultural trades, and for whom reports (either anecdotal or published) exist regarding the SARS-CoV-2 virus and/or the resulting disease state COVID-19. Available literature was searched using an artificial intelligence (AI)-assisted engine, as well as via common databases, such as Web of Science and Medline. The present review focuses on susceptibility and transmissibility of SARS-CoV-2, and polymorphisms in transmembrane protease serine 2 (TMPRSS2) and angiotensin-converting enzyme 2 (ACE2) that contribute to species differences. Dogs and pigs appear to have low susceptibility, while ferrets, mink, some hamster species, cats, and nonhuman primates (particularly Old World species) have high susceptibility. Precautions may therefore be warranted in interactions with such species, and more selectivity practiced when choosing appropriate species to serve as models for research.

## 1. Introduction

With the continuously growing threat of coronavirus disease (COVID-19) across the globe, the need for comprehensive research regarding infection and transmission of the causative virus, Severe Acute Respiratory Syndrome Coronavirus 2 (SARS-CoV-2), continues to increase, particularly as new variants of the virus emerge. Human welfare consequences of this global event are obvious, but there are a number of nonhuman animal welfare impacts as well. Among these are fears that nonhuman animals may be vectors for the virus that can accelerate its spread. In the immediate aftermath of the virus’ explosive spread in China, little was known about the nature of transmission, other than that Patient Zero was putatively associated with the Huanan Wholesale Seafood Market in Wuhan, China, where processed animal products and live animals are sold [1]. The idea that animals were associated with the spread of the virus was perhaps especially salient in an area of the world that in 2013 had seen a similar cross-species jump from chickens to humans that was the start of the (to date, localized) A(H7N9) avian flu, the causal agent of the H1N1 pandemic [2].

Ensuing panic regarding SARS-CoV-2 and nonhuman animals as possible vectors for the virus resulted in reports of pets being thrown from apartment windows to their deaths in some parts of the world [3], and some 17 million mink in Denmark were euthanized when a COVID-19 variant was discovered in several mink farms, both in mink and farm personnel [4]. Subsequent reports of companion animals, livestock, and zoo animals contracting COVID-19 from their human caregivers have been published [5], and these reports, though infrequent, make it clear that, the SARS-CoV-2 virus poses a risk to some nonhuman species similar to the risk to humans.

The crippling impact that COVID-19 has had on humans—both those immediately infected with the disease and those experiencing its adverse consequences on their lives and livelihoods—has also had an impact on nonhuman animal welfare. In the United States (USA), the number of unemployed rose from 6.2 million in February 2020 to 20.3 million just 3 months later [6]. Animal surrenders to shelters increased as people found themselves less able or willing to care for their animal companions [7,8]. As multiple COVID-19 outbreaks appeared among USA meat packing personnel, food-processing plants shut down and a number of animals produced for human consumption that could no longer be sold faced euthanasia, sometimes by inhumane means [9].

The dire need for accelerated development of a safe and effective vaccine has put tremendous pressure on researchers to identify appropriate nonhuman animal models, not only for use in vaccine development, but in expanding our understanding of COVID-19 and its possible treatments. The ethical framework for humane laboratory animal welfare known as the “three R’s,” first outlined by Russell and Burch in 1959 [10], compels us to be thoughtful and deliberative in our use of nonhuman animals as research subjects; to replace them with alternatives when possible, to reduce the numbers of animals used, and to refine the kinds of studies that employ animals in ways better guaranteed to produce informative results and, thus, justify to some extent the cost to animal lives. If researchers are to better understand SARS-CoV-2 and related coronaviruses, develop more effective vaccines, prevent infection, and determine more effective treatments for those already infected, then it is imperative not only from an animal welfare perspective, but for the benefit of human welfare as well, that moving forward the animal species best suited to be effective research subjects are chosen for study.

While we typically think of SARS-CoV-2 in terms of its devastating impact on human welfare, it is clear that the virus has had and continues to have an adverse effect on animal welfare. In addition to human caregivers’ compromised abilities to care for their animals, animal welfare is compromised by the vulnerability of animals to the virus, their potential as unintentional vectors, and their abilities to serve as effective research models for the disease as it manifests in humans. In this paper, we review what is currently known regarding factors that appear to affect susceptibility to the SARS-CoV-2 virus in a select sample of terrestrial nonhuman mammalian animal species, and the ability of animals to act as possible vectors.

Literature for the review was collected using manual curation and a natural language AI-powered search engine created by Dr. Tayab Waseem at Eastern Virginia Medical College. The AI engine identified all SARS-CoV-2 literature published up to 15 June 2020 that addressed the question, “What is the risk posed by common domestic animals to humans regarding transmission of the SARS-CoV-2 virus?” Subsequent species-specific searches utilized databases such as Web of Science, Scopus, and Medline. The species and taxa selected for review here were chosen due to the potential frequency of their human contact, and the frequency with which their mention was appearing in the most recent literature on COVID-19. Our focus is on terrestrial mammals. The important topic of COVID-19′s potential impact on marine mammals, given that this is a respiratory disease of air-breathing mammals, we must leave for other investigators. Thus, our review is not meant to be comprehensive, but rather is intended to aid with summarizing relevant literature to accelerate research with the goal of improving our understanding of the SARS-CoV-2 virus, and the identification and reduction of adverse welfare impacts of this virus on nonhuman mammals, particularly those with close human contacts.

### 1.1. Potential Animal Welfare Impacts of the COVID-19 Pandemic

The impacts of human behavior on nonhuman animal species vary in scope and form, from impacts derived by anthropogenic climate change, habitat destruction, and pollution, to more direct effects experienced as a consequence of the food industry, fur, entertainment, and pet trades. With respect to the current pandemic, most of these human impacts have been altered—some in ways that benefit nonhuman animals, and some that are to their detriment.

Among the benefits were a noticeable drop in anthropogenic noise [11,12] and other forms of pollution [13,14,15,16], as well as vehicular traffic and air travel [17], as countries mandated lockdowns in an effort to control the spread of the SARS-CoV-2 virus. These changes may have provided many species with some relief from human-generated pressure. Some reported a return of wildlife into urban areas, as indicated by increased sightings of such species [18,19], though others have suggested that such sightings are nothing more than an increase in observations of species that had always been present [20]. One study reported a significant decline in road mortality in wild hedgehogs correlated with the global shut down and resulting decreased motor traffic [21].

Another potential welfare benefit pertains to a decrease in euthanasia of shelter-surrendered companion animals, concomitant with an increase in adoption rates during the pandemic [22,23,24] as people facing mandated social isolation for unknown periods of time sought nonhuman company. China also reclassified dogs as “pets,” rather than as livestock, in a move towards reducing consumption of these animals [25]. Wildlife species may also have benefited from the pandemic; as news spread of a so-called “wet market” [26] being the virus’s putative origin, such markets were temporarily banned by the Chinese government [27], consumer demand for exotic wildlife declined [28], and trafficking of wildlife also declined, although only temporarily [29]. At the same time, however, some wildlife research and rehabilitation organizations were called upon to slow or cease their engagement with wildlife populations. In Canada, for example, the Canadian Wildlife Health Cooperative called for a temporary halt to all bat rehabilitation and research programs [30]. Stops such as these to wildlife monitoring, research, and assistance programs as a consequence of the pandemic pose as obvious welfare threats to wild species. The anthropause resulting from the pandemic also impacted wildlife distribution in ways that might have a deleterious effect on some species. Torresian crows (*Corvus orru*) in Australia, for example—a species that normally scavenges for human foodstuffs in urban areas—were found in significantly greater abundance on beaches, where some researchers fear they might be outcompeting and depredating the nests of other resident species [31]. The shift in the crows’ foraging habits was attributed to the substantial decrease in human-generated trash available in urban areas in the wake of pandemic shutdowns.

Among the detriments to animal welfare that may be linked directly or indirectly to the COVID-19 pandemic have been acts of violence threatened or actually committed against animals as putative causal agents of the disease’s spread [32]. As noted above, in the initial spread of the virus, its nature of transmission was unclear, and there some concern about the possibility that pets might serve as carriers. The international press reported stories of pets being abandoned in the streets of Wuhan after China’s lockdown of that city, and of some animals purportedly being thrown to their deaths from apartment windows [3]. As the Chinese government strove to control the spread of disease, thousands of people were forced to evacuate their homes and leave their pets behind, putting these animals at risk of starvation [33]. This same source reported that some organizations in China announced a campaign to kill any cats or dogs found outdoors, in an effort to prevent disease transmission. While it is unlikely that pets were the original zoonotic reservoir for the disease, it does appear possible for humans to transmit the virus to some other animal species. A recent study found 4 of 114 stray cats captured in Zaragoza, Spain to be seropositive for SARS-CoV-2 [34]. Some researchers speculated that the severity of the COVID outbreak in northern Italy might be due to high rates of dog ownership in that part of the country [35], and others argued that the few data demonstrating SARS-CoV-2 infection in pet dogs and cats, along with the known similarities between humans and some common domestic species in ACE2 receptor binding sites (as reviewed in this article and elsewhere) demand a serious consideration of pets as possible sources for disease transmission [36]. Professional opinions such as these may contribute to public fears about animals as potential disease vectors and concomitant pet abandonment or violence directed towards common domestic species. Separately, conservation groups have also worried about the potential slaughter of wildlife by people fearful of disease transmission [37], or seeking retribution for the pandemic in some way.

Indeed, some nonhuman animal species have tested positive for the SARS-CoV-2 virus including some domestic dogs and cats [38,39,40,41] reportedly contracting the virus from their human caregivers. In addition, five captive Malayan tigers and three lions in the Bronx Zoo in New York City (USA) and several gorillas in the San Diego Zoo in San Diego, CA (USA) have tested positive for the virus, reportedly contracting it from infected zookeepers [42,43,44]. Farmed mink have also tested positive for COVID-19, and mink-to-mink, human-to-mink, and mink-to-human transmission has been determined [45]. More than 1 million mink in countries such as Spain and the Netherlands have been euthanized as a result of reports such as these [46]. In the USA, one wild mink in Utah has tested positive for SARS-CoV-2 [47]. Similarly, two wild mink in Spain have been documented with SARS-CoV-2 infection phylogenetically identical to the variant originating in Wuhan [48], thus demonstrating the virus’ ability to jump to wild populations of species with high susceptibility to the virus. One worry is that feral animals may carry the virus into wild populations, or become a reservoir themselves for the virus; in one study, all 11 feral mink presumed to be escaped from the mink farm in Utah near where they were caught were seropositive for SARS-CoV-2 [49]. The potential for the virus to become established in wild animal populations is a welfare threat not just to those animals but to humans as well, to the extent that such an establishment creates a reservoir for the virus.

Other consequences of the pandemic that have been detrimental to animal species are more indirect, and stem from the consequences of attempts to control viral spread, government lockdowns, subsequent economic losses, and personal illness. Indiscriminant use of disinfectant to help control the virus has led to the deaths of wildlife in China [50], for example. While increases in pet adoptions from shelters have been reported as people seek nonhuman animal companions during mandated and self-imposed social isolation (ex. [24,51,52]), other sources report such upticks as resulting only from an increase in volunteers fostering, while actual adoptions during the pandemic have declined [53]. Such declines may be a reflection of changes in financial security resulting from shelter-in-place and similar mandates. An increase in animal surrender to shelters may result for similar reasons. Within the shelter environment, fewer employees on site and fewer volunteers available to care for surrendered animals may also result in some compromise of welfare.

Similarly, the shortage of caregivers has resulted in the premature euthanasia of many lab animal species housed in research facilities around the world [54], and the lack of volunteers and reduction of employees engaged in conservation work has increased the activities of poachers and wildlife traffickers, even as public demand for exotic animal goods waivers in the uncertainty of the pandemic’s putative source [28].

In the home, the increased time spent with human companions no longer leaving the house for work each day may be enjoyed by some pets, but also a potential source of stress as disruption of previous routine [23]. Loss of income, fear of illness, insecurity about food and housing, and restricted movement are additional stressors on human caregivers that may increase household violence [55] of which pets may be unfortunate victims. Actual illness of caregivers can also compromise animal welfare, especially if caregivers are removed to hospitals or succumb to their illness, leaving their animal charges, perhaps, without adequate care. Indeed, loss of income, forced evictions, and personal illness have all contributed to increases in animal abandonment in some parts of the world [56].

In the USA, some of the highest rates of human infection with SARS-CoV-2 have been among personnel working in meatpacking and other food processing facilities, resulting in large numbers of employees becoming ill at the same time and consequential facility closures. Some estimates are that as many as 334,000 of the COVID-19 cases in the USA at the time this paper was being written were among such personnel [57]. Such closures have resulted in livestock animals spending longer times in transport and holding, and at handling facilities, and in some cases, delays have led to decisions by livestock owners to mass euthanize their stock rather than continue to support animals that cannot be received at slaughter and packing plants [58]. Similar livestock animal welfare challenges were experienced elsewhere in the world; in Australia, for example, nearly half of the human crew for a livestock carrier ship arriving to transport a shipment of 56,000 Australian sheep to the Middle East tested positive for COVID-19, resulting in a delay of the shipment [59,60].

Finally, animals may experience stress and compromised wellbeing because their human caregivers are experiencing the same. Dogs are susceptible to emotion contagion with their human caregivers [61,62,63] and, thus, may experience distress, as their human caregivers are distressed. Horse owners in the United Kingdom expressed concern about their inabilities to ride or interact with their horses and the likely negative effects that such reduced interactions might have on those animals [64]. In yet another article, reduced interactions with zoo visitors as a consequence of mandated lockdowns was reported to increase vigilance and human avoidance in some zoo animal species, and increase human proximity-seeking in others [65]. Taken together, these data suggest that the current pandemic, in addition to its grave effects on the welfare of humans, may have significant impacts on nonhuman animal welfare. At least in part, these welfare impacts are influenced by not only the pandemic’s effects on humans, but by perceived or actual susceptibility and transmissibility of SARS-CoV-2 to humans and other animals. In the rest of this manuscript, we review what is known about such susceptibility among several mammalian species commonly in contact with humans.

### 1.2. Predicting Susceptibilities: TMPRSS2 and ACE2

Among the factors that influence the contagiousness of SARS-CoV-2 are the polymorphisms in genes coding for the receptor in animal cells to which the virus binds. Polymorphisms are genetic code variations shared by many individuals and can confer evolutionary advantage or disadvantage through natural selection. In regard to the novel coronavirus, there are two mammalian proteins that are key to viral susceptibility: angiotensin-converting enzyme 2 (ACE2) and transmembrane serine protease 2 (TMPRSS2) [66,67]. ACE2 is a transmembrane protein on lung cell and other tissue surfaces that acts as the SARS-CoV-2 viral receptor and allows viral endocytosis [67,68]. Although the specific effects of TMPRSS2 on SARS-COV-2 are not fully understood, it is thought to activate the virus’ characteristic spike protein as a requisite step for endocytosis [69]. Since polymorphisms for ACE2 and TMPRSS2 exist in multiple species [70,71,72,73,74], the binding affinity of SARS-CoV-2 for particular strains of ACE2 [71], combined with particular abundances and phenotypes of TMPRSS2 on lung cells [75], leads to variation of viral susceptibility across species. The degree to which nonhuman animal polymorphisms for ACE2 and TMPRSS2 resemble those in humans, therefore, is likely to predict indirect and direct transmission of the SARS-CoV-2 virus and subsequent presentation of the disease COVID-19.

Recent studies have shown the transmissibility of SARS-CoV-2 from one species to another varies. The basis for this variation is at least in part due to the ease with which the virus’ envelope spike proteins can bind to the polymorphic ACE2 receptor [70,71]. These variations in functionality are dependent not only on the presence and activity of TMPRSS2 [75], but also on the interactions of the spike protein with specific amino acids that make up the ACE2 receptor [66]. Between 62% and 99% of the amino acid sequence for the ACE2 receptor is conserved across at least 23 mammalian species, including humans, dogs, cats, pigs, cattle, sheep, horse, and ferrets [71]. Within the ACE2 primary sequence, four key amino acids (K31, Y41, N90, and K353) have been found to correlate with an increased susceptibility to SARS-CoV-2 infection [70]. Two of these amino acids, K353 and Y41, are two of ten known binding sites on the human ACE2 receptor for the SARS-CoV-2 virus [72]. The correlation between the K353 and Y41 sequences and increased susceptibility to SARS-CoV-2 could be explained by the similarity of ACE2 binding site polymorphisms containing K353 and Y41 across mammalian species. Species carrying polymorphisms that are compatible at all four key amino acids (K31, Y41, N90, and K353) most likely represent species with the highest possibility of viral susceptibility [73]. Table 1 outlines genetic polymorphisms of the ACE2 receptor in twelve mammalian species.

## 2. Nonhuman Animal Transmission and Susceptibility

Below, we review what is known about susceptibility to and transmission of COVID-19 (as well as similarity of symptoms) in mammalian species most likely to come into contact with humans through their utilization as companion, research, or farm animals. Table 2 outlines reported susceptibility, disease symptom presentation, and predicted transmissibility to humans of ten mammalian species, including several (bats and pangolin) suspected as original vectors for the jump to humans that triggered the current pandemic. Not all of the species reviewed in the text that follows appear on this table.

### 2.1. Nonhuman Primates

Due to their close evolutionary relationship to humans, nonhuman primates (NHPs) are commonly employed as research subjects in studies of human pathologies. According to the United States Department of Agriculture’s (USDA) annual report [92] from Fiscal Year 2017 (the most recent year for which data were available), 75,825 NHPs were utilized in registered research facilities. NHPs also comprise a large percentage of the species housed in zoological parks—22 of the 300 species homed at the San Diego Zoo in California, for example, are monkeys [93]—and NHPs are reported by visitors to be one of the most popular zoo species [94,95]. Ecotourism ventures that bring tourists to see NHPs in the wild are also increasingly popular, and the economic potential of these ventures makes them an attractive option to many nations with wild populations of NHPs. Thus, the opportunities for transfer of disease between humans and NHPs are many, and human diseases remain a significant welfare risk to NHPs in locations where there is close proximity of NHPs to humans [96]. Indeed, research suggests that respiratory viruses contracted from humans are the leading causes of death among wild chimpanzee (*Pan troglodytes*), bonobo (*Pan paniscus*), and mountain gorilla (*Gorilla berengei berengei*) populations in Africa [97].

With respect to COVID-19 susceptibility in NHPs, orthologues of human TMPRSS2 can be found across many NHP species [98]; among humans, polymorphisms in TMPRSS2 have been postulated as an explanation for global population differences in COVID-19 infection rates and disease severity [99,100]. With regard to ACE2 polymorphisms, the apes (Hominidae) and Old World monkeys (Cercopithecidae) share with humans 12 amino acid residues key to recognition by the SARS-CoV-2 receptor binding domain [101], suggesting that NHP species from these families may be particularly susceptible to the SARS-CoV-2 virus. The conserving of amino acid morphs in species that share a closer evolutionary history is not surprising, and with a few exceptions, most of the NHPs identified as most at risk in Melin et al.’s [101] review are those whose ancestral deviations from our own evolutionary path are less distant in time.

The actual risk of COVID-19 to NHPs is still being determined, and at present consists of a single report in some zoo gorillas (described below), in conjunction with some data derived from research using NHPs as subjects that has been aimed at understanding the disease and identifying putative therapies. It should be noted that for most if not all of the studies reviewed below, data are based on a small number of individuals and have (in the interest of accelerating dissemination of knowledge), been published without peer review.

An appropriate nonhuman animal research subject for COVID-19 should ideally be one in which the disease manifests in the same way that it does in humans. Perhaps because of their obvious evolutionary relatedness to humans, Old-world NHPs were in use as research subjects in SARS-CoV-2 studies as early as March 2020 [102]. In that study, eight cynomolgus macaques (*Macaca fascicularis*) were inoculated both intratracheally and intranasally with virus obtained from a confirmed human case. With the exception of one animal that showed excessive nasal discharge on post-inoculation day 14, none of the monkeys showed any clinical symptoms. However, in a study by Guebre-Xabier et al., 2020 employing the same species, SARS-CoV-2 exposed animals showed moderate to severe lung pathologies, consistent with those seen in human subjects [103]. A more recent study comparing infection susceptibility and symptoms in rhesus macaques (*M. mulatta*) and *M. fascicularis* showed similarly mixed results, with both species showing elevated body temperatures, but only *M. mulatta* showing decreased activity levels. Both species showed pulmonary lesions in CAT scans, and viral shedding via the respiratory system [104]. It is not clear why these studies should yield such different results, though in all of them the number of subjects employed was very small.

A similarly mixed set of results with respect to clinical appearance of symptoms is available for rhesus macaques (*M. mulatta*), another NHP species typically used in laboratory settings. In one early study (appearing in May 2020), Bao et al., 2020 [105] investigated whether prior exposure of *M. mulatta* to the virus provided any immunity to later re-exposure. In that study, six rhesus macaques served as subjects inoculated with the virus intratracheally on two occasions, 28 days apart. Of the six, only one showed clinical symptoms similar to those seen in humans (pneumonia), though antibody production did increase in all six subjects by day 15 post-inoculation. In another study, however, intratracheal inoculation with SARS-CoV-2 resulted in severe respiratory symptoms in all eight animals in the study, particularly in animals over the age of 16 [106]. Symptoms similar to those seen in humans—but again, less severe—were observed in eight *M. mulatta* employed as subjects by Munster et al., 2020 [107].

Comparing pathology in response to SARS-CoV-2 inoculation in six individuals from each of two Old World (*M. mulatta* and *M. fascicularis*) and one New World species (*Callithrix jacchus,* the common marmoset), only members of the Old World species showed symptoms of disease [108]. Laboratory investigations employing another Old World species, the African green monkey or grivet (*Chlorocebus aethiops),* have shown successful infection with a much lower dose of the virus and a severity of symptoms similar to that seen in humans [109,110], suggesting that this species may be optimal for investigations of SARS-CoV-2 with the aim of advancing our understanding of the nature of the virus as it affects humans. Others have shown that both *M. mulatta* and *M. fascicularis* show symptom severities similar to those in humans with mild to moderate disease [111]. Similarly, a comparative study aimed at determining sensitivities to airborne transmission of the SARS-CoV-2 virus detected viral shedding and respiratory symptoms in a small group of *M. mulatta, M. fascicularis*, and *C. aethiops* exposed to the virus through a controlled aerosol exposure delivered to the head [112]. Symptom severity was generally mild, however, and varied across species. Again, these studies suggest that Old World NHP species may be more susceptible to the virus than New World species.

World-wide, unintentional infection of NHPs with SARS-CoV-2 remains a significant concern for many conservationists, particularly those who work in areas subject to increasing human-animal conflict and those where ecotourism involving NHPs is common [113]. Several recent outbreaks of respiratory diseases (other than COVID-19) in wild primate populations (see Spelman et al. [114] for a partial review) serve as poignant reminders of the welfare risk that human disease can pose to NHPs. While no reports of COVID-19 in wild NHP populations were found at the time of this manuscript’s preparation, this is not true of captive populations. In January 2021, the San Diego Zoo Safari Park reported that all eight of its Western lowland gorillas (*Gorilla gorilla gorilla*) were infected with SARS-CoV-2, and showing clinical symptoms of COVID-19 (coughing and congestion) [5]. This finding resulted in the subsequent inoculation of four orangutans (*Pongo* sp.) and five bonobos (*Pan paniscus*) at the San Diego Zoo with an experimental variant of vaccine developed for the veterinary trade by Zoetis [115].

### 2.2. The Mustelidae: Ferrets and Mink

Animals of the mustelid family, including domestic ferrets (*Mustela furo*) and American mink (*Neovison vison*), come into contact with humans through the pet and pelt trades. The domestic ferret is now a common companion animal; the American Veterinary Medical Association estimated 326,501 pet ferrets in the USA in 2018 [116], and reports ferrets as the third most popular pet animal in that country after dogs and cats.

With respect to mink, Humane Society International reported 60.3 million mink farmed for their fur worldwide in 2018 [117]. Farmed mink are typically maintained under husbandry conditions in which they are housed in close proximity to one another. Such close contact with one another and with human caretakers has led to outbreaks of COVID-19 at over 200 mink farms in Europe [118] and resulted in the subsequent preventative euthanasia of millions of animals in Denmark [119] and the Netherlands [120]. Similar outbreaks have also been documented in the USA, Sweden, Spain, Italy, and Greece [121], and human-to-mink, mink-to-mink, and mink-to-human transmission has been reported [122]. At least one study of mink-to-human viral transmission suggests that the virus underwent detectable spike mutations in mink hosts [123], raising concerns about the abilities of vaccine development to keep pace with viral mutations occurring in animal reservoirs. Thus, mustelid exposure to the SARS-CoV-2 virus is a definite welfare risk for these animals as well as for their human caretakers.

There is limited research on mustelid susceptibility to SARS-CoV-2 and how variations in TMPRSS2 may affect this. One recent review paper noted that at least one of the many protein sequences in TMPRSS2 that has been identified as important in the protease’s facilitation of SARS-CoV-2 entry into human cells shows a number of polymorphisms in ferrets, which may explain some of why this particular mustelid species is not as affected by the virus as is its mink cousin [67] (as discussed below).

Other studies, however, point to similarities in ACE2 receptor constituents between mustelids and other species (especially humans) as a factor that may predict mustelid sensitivity to SARS-CoV-2. In one, 92% of the amino acid sequences involved in production of ACE2 receptors in canines were also found in mink [74]. However, the differences between canine and mink amino acid sequences involved in ACE2 receptor binding domains may be sufficient to block SARS-CoV-2 entry into the cell, since the two species have shown some differences in susceptibility to this virus (see Section 2.6 below). In their review, Hancock et al. [67] also constructed phylogenetic trees based on similarities in protein sequences composing both the ACE2 receptor and TMPRSS2 and found that the species that shared the most amino acid sequences for this receptor and protease were also the species that to date have tested positive for SARS-CoV-2 (e.g., ferrets, mink, cats, tigers, and dogs).

Devaux and colleagues [70] showed that the ferret ACE2 receptor binding domain has two of the four key amino acids (K31 and K313) required for optimal binding by SARS-CoV-2. Devaux et al.’s report (as well as others described elsewhere in this paper) indicates support for the hypothesis that species with K31 and K353 amino acid components in their ACE2 receptors are more susceptible to SARS-CoV-2, and that such similarities across species may be a good predictor of species susceptibility.

However, in vivo studies involving inoculation of ferrets with the virus suggest that this susceptibility is not always demonstrated in obvious clinical symptoms nor in viral transmission. In one such study [124], two ferrets were inoculated intranasally with the virus, and then pair-housed with a naïve conspecific. For each of the inoculated animals, a third ferret was also housed in indirect contact by means of a partition between cages that allowed airflow, but no direct contact. All animals were monitored for symptoms of disease. Infected ferrets showed reduced activity and some coughing, but no weight loss or mortality. Of the exposed uninoculated (“naïve”) animals, only those in direct contact with infected individuals showed increased body temperatures, but no concomitant weight loss. Infectious virus was detectable in nasal washes of infected animals on day 2 post-inoculation through day 8 post-inoculation. In a similar study but one that obtained very different results, Richard et al. [125] inoculated 4 ferrets intranasally with SARS-CoV-2, and housed each with an naïve conspecific in the same cage, as well another naïve animal in a nearby cage separated from the cage with the infected animal by two steel grids, 10cm apart. Throat, nasal, and rectal swabs were collected on alternating days and assessed for the presence of the virus. In inoculated animals, SARS-CoV-2 RNA levels peaked 3 days post-inoculation, and were detectable in at least one animal up to 19 days post-inoculation. All direct contact animals were infected by their inoculated cagemates, and three of the four indirect contact animals also tested positive for the virus. The authors do not present data on observed clinical symptoms, if any.

Shi et al. [77] inoculated intranasally each of three groups of ferrets with one of three strains of SARS-CoV-2. Animals were euthanized on post-inoculation day 4, and tissue samples collected from the respiratory and gastrointestinal tracts, brain, sinuses, and several other body areas. Using PCR techniques, viral RNA and infectious virus were found in the nasal turbinates, soft palate, and tonsils of inoculated animals, but were not detected in any other tissue. A subsequent study reported in the same manuscript inoculated intranasally six ferrets, and housed each in an isolator. Rectal and nasal swabs were taken on days 2, 4, 6, 8, and 10 post-inoculation and assessed for viral presence. In addition, animals were monitored for any sign of disease. Viral RNA was detected in nasal washes in all animals on all sample days, but only a few showed viral RNA in rectal samples. Infectious virus was detected in the nasal washes of all subjects, but in none of the rectal samples. Only two of the ferrets displayed clinical symptoms (appetite loss and fever). Antibodies against SARS-CoV-2 were detected in all inoculated animals. These data suggest that while ferrets are susceptible to infection with SARS-CoV-2, they do not appear to be as adversely affected by it as are humans nor (as discussed below) as their mink cousins.

The studies described above utilized laboratory ferrets, but one group of researchers assessed rates of SARS-CoV-2 infection in pet ferrets living with their human owners. Giner et al., 2021 [126] collected blood samples from 127 pet ferrets in Valencia, Spain. Ferrets were recruited from among patients seen at a single veterinary clinic for routine care or medical problems. Of the 127, only two were seropositive for SARS-CoV-2. Samples were collected in June of 2020, at the end of Spain’s government-mandated lock down during which citizens were required for the most part to stay at home. At that time, human COVID-19 case rates in Spain had dropped to an average of 450 cases per day [127]. In a similar study, oropharyngeal and rectal swabs were taken from 71 ferrets used in rabbit hunting in Ciudad Real, Spain. Using an qRT-PCR assay, SARS-CoV-2 RNA was found in only 6 animals, and the virus was only isolated from one rectal swab. The authors conclude that SARS-CoV-2 infection can occur in ferrets when its circulation among sympatric humans is high, but that small populations of ferrets do not seem as capable of maintaining viral circulation, as do other larger mustelid populations, such as what are found on many industrial mink farms [128]. It is not yet clear why ferrets can become infected but yet show few symptoms, nor to what degree, if any, ferrets pose a possible threat to humans as a reservoir species for SARS-CoV-2.

The susceptibilities of mink (*Neovison vison*) to the virus appear to be greater than they are for ferrets, though presentation of symptoms (as in humans) varies. Clinical symptoms of SARS-CoV-2 infection observed in a study of mink on Danish fur farms, for example, ranged from decreased feed intake to nasal discharge to severe respiratory distress and mortality [129]. In a study of laboratory-inoculated mink, Shuai et al., 2021 [130] revealed replication of the virus in the upper and lower respiratory tract that caused lung lesions similar to those in seen in humans with COVID-19.

Oreshkova et al., 2020 [118] evaluated mink at two farms in the Netherlands and reported that the most common symptom was nasal discharge, but that some animals did show severe respiratory distress. Necropsies of symptomatic animals that died during this time revealed interstitial pneumonia as well as lung lesions. Viral RNA was also detected in lung tissue, throat swabs, and rectal swabs taken from these animals. The same study documented what appeared to be mink-to-farm worker transmission, based on gene sequencing data obtained from viral samples collected from the infected mink and humans [45]. Suggested routes of infection to mink and to humans in the fur farm environment included exposure to infected humans or fomites contaminated by them, to airborne droplets expelled by humans or mink, or to dust from contaminated bedding handled by naïve humans or exposed to naïve mink. Oreshkova et al. collected dust samples at the two farms they monitored and obtained positive tests in those samples for viral RNA. Several stray cats who wander the farm grounds also tested positive for SARS-CoV-2 antibodies, suggesting the cats could have become infected due to exposure to the minks. Data such as these suggest that mink may be a possible reservoir for the SARS-CoV-2 virus [131], and that greater care should be taken with respect to husbandry hygiene to reduce possible viral transmission.

### 2.3. The Suidae

The Suidae are another mammalian family that may be a vector for the virus SARS-CoV-2. Domestic pigs (*Sus scrofa*) are relevant to study because of their close proximity to humans through their use as a food animal, and increasingly (particularly for the smaller breeds) as pets. They are also a commonly used research species, especially in biomedical research [132]. For instance, because of similarities between human and swine cardiovascular systems, pigs are commonly used animals in cardiovascular research [133], and one laboratory research line (the obese Ossabaw pig) has been promoted as a good model for the differential impact of COVID-19 on humans with comorbid obesity [134].

Because pigs are highly susceptible to experimental and natural infection by a related betacoronavirus (SARS-CoV) [135], there is concern regarding the ability of the Suidae to transmit the virus to humans. Structural analyses of the binding domain of porcine ACE2 receptors also suggest that pigs would be susceptible to SARS-CoV-2 [136,137]. Meekins et al. further demonstrated that the virus could replicate in swine cell lines [138]. However, at least one in silico study showed that the receptor-binding domain of the SARS-CoV-2 virus could not recognize ACE2 receptors in pigs [139]. With respect to the role that TMPRSS2 may play in swine susceptibility to SARS-CoV-2, little was found by the present authors regarding that protease in this taxonomic group.

Pigs have been shown to be susceptible to other coronaviruses, including SARS-CoV, the virus responsible for a 2003 outbreak of Severe Acute Respiratory Syndrome (SARS). However, of 242 animals tested from a region with a major human SARS outbreak, only 2 tested positive for viral antibodies in serum samples evaluated using ELISA and Western Blot assays [76].

Both SARS-CoV and SARS-CoV-2 have been found to replicate in porcine cells *in vitro*, raising concerns for possible transmission of the SARS-CoV-2 virus through human consumption of pork products rather than from exposure to living animals [138,140]. In the Meekins et al. study, intact living animals showed no clinical symptoms subsequent to inoculation with SARS-CoV-2 [138]. Other researchers have also been unsuccessful in inoculating swine with SARS-CoV-2 [77,141].

More recently, however, another research group did succeed in inoculating pigs with SARS-CoV-2 [142]. In this study, 16 Yorkshire crossbred pigs were oronasally inoculated with a viral dose 10 times that used in the previous studies mentioned above. Animals were then housed together in two groups of eight. Starting at inoculation and every other day thereafter, blood samples were collected, along with nasal washes, and rectal, nasal, and oral swabs. Oral fluids were collected from chewing enrichment toys in the animals’ enclosures, and necropsies were performed on sample pigs on days 3, 22, and 29 post-inoculation. Symptoms included ocular and nasal discharge, but only for the first 3 days post-inoculation. No respiratory symptoms were detected other than a mild cough in one pig, and no elevated temperatures were observed. SARS-CoV-2 RNA was detected in the nasal washes of two pigs by day 3 post-inoculation, using qRT-PCR. No live viral shedding was ever detected [142]. The relative lack of successful inoculation of pigs with SARS-CoV-2 suggest that pigs are unlikely to serve a useful role as research animals for investigations of SARS-CoV-2 or for the disease state COVID-19.

Nonetheless, the COVID-19 pandemic has had an adverse effect on domestic pig welfare, primarily through its effects on the food industry in the form of substantive outbreaks of the disease in meat packing plants, both in the United States and in Europe [58]. The number of workers at these plants sickened by the SARS-CoV-2 virus resulted in widespread closures and a concomitant back up in the timely shipping, slaughter, and processing of animals that in some cases, appears to have led to the euthanasia of some pigs that were not able to be brought to market [143]. Indeed, the National Pork Board in the USA, anticipating a crisis in stockyards and holding facilities unable to send animals on to meat processing plants, hosted webinars on humane emergency depopulation methods [144], and some agricultural officials estimated that upwards of 700,000 pigs were likely to be euthanized because of slaughterhouse closures [145]. Such unwarranted animal slaughter on such a large scale is taxing on personnel charged with this task [146], and adds to the stress already likely experienced as a consequence of illness or unemployment among such workers due to the pandemic. Livestock personnel under similar duress have been show to delay decision-making in ways that compromised swine welfare [147], suggesting yet another possible negative welfare impact that the COVID-19 pandemic may have had on this taxonomic group.

### 2.4. Other Hooved Species

Among the many nonhuman animal species regularly in contact with humans over much of the world are hooved stock such as sheep, goats, horses, pigs, llama, alpaca, camels, and deer. Many of these animals are raised for human consumption in one form or another—as food products, or producers of dairy products or wool. Some are used as work animals, others for entertainment or as companions. Around the world, for instance, an estimated 111.800 million equids (horses, donkeys, and mules) serve humans as pack and dray animals, as pets, transport, or show animals, tourist attractions, or as producers of milk and meat [148]. The same source estimates a global count in 2019 of over 1.510 million cattle, 1.238 million sheep, 1.100 million goats, 37.5 million camels, over 9.15 million camelids (alpaca, llama, and related species), and over 204,000 buffalo. As with the equids, these other species also play important roles in human lives as producers of food and fibers, as well as transportation and providers of labor. The consequences of a virus such as SARS-CoV-2 infecting common domestic hooved stock such as these species would be profound, for both human and nonhuman animals.

As is the case for the other species reviewed in this paper, coronaviruses of one sort or another are common causes of illness in ungulate species. Many of these viruses are enteric and present with gastroenteritis ranging from mild to severe in nature, though some are systemic and include respiratory symptoms more like those seen in response to SARS-CoV-2 [149]. Across the ungulates, the proteins composing ACE2 receptors appear to be relatively conserved, with some variations at the binding site for SARS-CoV-2 that appear to predict sensitivity to this particular coronavirus variant (see below). As reviewed elsewhere in this manuscript, expression of ACE2 in the lungs and functionality of TMPRSS2 as a protease facilitating viral entry also varies between species. On the basis of similarities to humans in the amino acids comprising the receptor binding domain of ACE2 receptors that is engaged by SARS-CoV-2, some research suggested that domestic horses (*Equus caballus*), domestic cattle (*Bos taurus*), and domestic sheep (*Ovis aries*) are among the ungulate species whose ACE2 receptors might bind the virus, while the dromedary camel (*Camelus dromedarius*) was unlikely to show such binding [78].

In another study, ACE2 receptor protein sequences were compared across 410 vertebrate species and results were rated with respect to likely binding of SARS-CoV-2 as “very high,” “high,” “medium,” ‘low,” or “very low” based on the propensity of the virus’ binding to those protein sequences. With respect to ungulate species, three cervids were rated as having ACE2 receptor sequences with a high propensity for binding SARS-CoV-2, and 21 artiodactyls scored as medium (including domestic cattle, sheep, goats, and water buffalo) [150]. A similar study comparing ACE2 protein sequences in humans and 22 nonhuman animal species (including six ungulates) concluded that *C. dromedarius* and *O. aries* have a higher binding affinity to SARS-CoV-2 compared to humans, while alpaca (*Vicugna pacos*) have a binding affinity about equal to that of humans, and *E. caballus* and Przewalski’s wild horse (*Equus prezwalskii*) have lower binding affinities [151].

Not all of the many genetic analyses conducted to date have come to the same conclusions, however, perhaps in part because studies vary in which of the ACE2 receptor protein sequences they consider key to SARS-CoV-2 binding and cellular entry. In one study that included as part of its susceptibility ranking similarities in sequences that affect glycosylation at the binding domain, cattle (*Bos taurus*) and goats (*Capra hircus*) were rated as intermediate in susceptibility to infection, while horses (*Equus caballus*), dromedary camels (*Camelus dromedarius*) and Bactrian camels (*Camelus bactrianus*) were rated as high in susceptibility [152].

The above-mentioned studies were all based on statistical models applied to genetic analyses of receptor protein sequences. Experimental studies, in comparison, have yielded somewhat different results. In one using ex vivo tracheal and lung cell culture, for example, replication of SARS-CoV-2 was obtained in sheep and cattle samples, but not in swine samples [153].

In vivo studies attempting to inoculate cattle with SARS-CoV-2 demonstrate the difference between what gene sequence or tissue culture data might suggest and what happens in the living animal. In one such study, six dairy calves were intranasally inoculated with SARS-CoV-2 and housed 24 h later with three non-exposed conspecifics. Animals were monitored for clinical symptoms of infection, and blood, nasal, oral, and rectal swabs were also collected. Although tests of collected samples indicated viral replication in two of the inoculated animals, such replication was not sustained. None of the animals displayed clinical symptoms of disease, and the naïve conspecifics did not show any evidence of infection [154]. In a similar study, three colostrum-deprived Holstein bull calves were inoculated with SARS-CoV-2, either intratracheally or intravenously. Three additional calves housed in the same location but prevented from social contact with the infected animals served as control comparisons. Samples from all calves were collected at three points prior to inoculation and 12 points subsequently, and included nasal and rectal swabs, whole blood, and voided urine. One calf from the experimental group was euthanized on days 9, 16, and 21 post-infection, and tissue samples collected from a number of organ systems, including the respiratory and gastrointestinal tracts. Results across all assessments revealed no productive replication of the virus [155]. The same research group using similar methods, however, found that white-tailed deer fawns (*Odocoileus virginianus*) were highly susceptible to SARS-CoV-2 infection that was transmissible to other deer, although none of the infected animals showed any clinical symptoms [156]. The USDA’s website at the time of this manuscript’s preparation reported no incidents of ungulate infections in the USA [157].

Among the camelid species, dromedary camels (*Camelus dromedarius*) with prior exposure to Middle-Eastern Respiratory Syndrome (MERS) and who were seropositive for the MERS coronavirus had anti-MERS-CoV antibodies that showed cross-resistance with SARS-CoV-2 [158]. *C. dromedarius* has been identified as the intermediate host of MERS-CoV, though animals carrying the virus are asymptomatic [159]. One paper reported the potential utility of antibodies derived from another camelid, the llama (*Lama glama*) to bind to SARS-CoV-2 spike proteins and thus serve as a possible therapy or preventative for COVID-19 [160], but this theory was based exclusively on in silico analyses. Nonetheless, the idea that antibodies produced in the camelids and other species might be of help in development of a treatment or preventative for COVID-19 resulted in an increase in consumer demand for camel products in countries where camels are common livestock animals [161].

Whether or not hooved stock are susceptible to SARS-CoV-2, the global pandemic resulting from its transmission to humans has nonetheless been impactful with regard to their welfare. In the USA, for instance, as reported previously, COVID-19 hit meat processing and packing plants particularly hard. As these industries are designed for maximum and continuous throughput, any delay in one portion (for example, delays in slaughter and processing due to worker illness or government-mandated shutdowns) results in an immediate back up in other portions of the chain (such as breeding, transport, and holding) [58]. With slaughter for the market no longer operational, stocking densities increased which resulted in overcrowding and concomitant animal stress [58]. The result in some cases was a culling of otherwise healthy animals in order to reduce stocking pressures. As noted above, for instance, in the USA the National Pork Board made an emergency request for permission to “depopulate” swine stock levels in that country [162].

Human emotional distress in response to the pandemic has been substantial [163], and this distress may compromise equid welfare in a number of ways. Recent studies suggest that, as is true with dogs, horses are susceptible to emotional contagion from humans [164,165], and thus may experience distress if their caregivers are distressed. Emotional distress in human caregivers may also compromise livestock animal care. In one study of criteria used to determine timely euthanasia for compromised pigs, researchers found that negative emotional and mental states in stockpersons delayed decision-making and thereby compromised swine welfare [147]. Another study suggested that personal distress of racing staff as well as restrictions in allowed routines may have compromised the welfare of racing horses [166]. As horse owners experienced financial insecurities due to job layoffs or personal illness, concern rose that animal surrenders might increase for this species, anecdotally well-known for its expensive upkeep [167].

Disruptions in routine husbandry and human-horse interactions have also been expressed by horse owners as a possible welfare concern [64], and reduced exercise as horse owners engaged in less riding may have put horses at increased risk for obesity and related ailments [168]. These authors, however, also suggest that similarities of the human experience under lockdown with the experiences of most horses under human care may result in a post-COVID improvement in equine husbandry practices, as horse caretakers acquire greater sympathy with their horses’ experiences.

### 2.5. The Felidae

Domestic and captive wild cats come into contact with humans in a variety of ways, allowing for potential SARS-CoV-2 transmission from humans to cats or cats to humans. Domestic cats are the second most popular household pet after dogs [169], with an estimated 370 million worldwide. Common estimates are that in the USA. alone, an additional 60–100 million feral cats may be at large [170]. Captive wild cats are common zoo animals, with an estimated 10,000 big cats in captivity in the USA, for example [171]. Thus, the Felidae are another animal family group that may come into frequent contact with humans in one way or another, and be at potential risk during the current pandemic.

Because they share similarities with humans in regards to ACE2 receptor sequence and expression, COVID-19 disease presentation, and ability to transmit the SARS-CoV-2 virus, domestic cats (*Felis catus*) may be at particular risk during the pandemic, and are also a likely animal model for SARS-CoV-2 research. With respect to SARS-CoV-2 susceptibility, based on the two biological entities discussed earlier in this manuscript (the ACE2 receptor and the protease TMPRSS2)—at the time of this review, little information was found on the TMPRSS2 protein of either domestic or wild cat species. However, the domestic cat has all four key amino acid sequences of the ACE2 receptor protein that correlate with increased SARS-CoV-2 susceptibility [70], and the sequence identity overlap of the domestic cat ACE2 receptor with the human ACE2 receptor is 85.39% [172]. The same authors show that the Siberian tiger (*Panthera tigris altaica*) shows a similarly high ACE2 receptor sequence identity overlap with humans of 85.77%. A structural analysis identified 20 residues of the human ACE2 receptor that contact the SARS-CoV- 2 spike protein receptor binding domain; members of the *Felidae* share 16 out of the 20 contacting residues [173]. Another structural analysis of the ACE2 receptor identified 13 binding domain contacting residues of the human ACE2 receptor; domestic cats share 12 of these 13, and they also share with humans 14 of the 15 hydrogen bonds at the SARS-CoV-2 RBD/ACE2 interface [174]. A third study reported 14 human ACE2-contacting residues with both domestic cats and tigers sharing 11 of the 14 [175]. High similarity between feline and human ACE2 receptors indicates a high probability that SARS-CoV-2 will bind effectively to feline ACE2 receptors.

Another similarity between human and domestic cat ACE2 is frequency of expression of the receptor in the gastrointestinal tract; tigers (*Panthera tigris*) share this similarity as well [176]. ACE2 is also highly expressed in the skin, ear tip, lungs, and retina of the domestic cat [177].

The first reported case of a domestic cat testing positive for SARS-CoV-2 was in March 2020 [84]. House cats, lions (*Panthera leo*), tigers, and a snow leopard (*Panthera uncia*) have tested positive for the virus [83,115]. In most of these cases, the animal had confirmed exposure to a human who tested positive for or was suspected to have COVID-19. In Hong Kong, 6 out of 50 cats living in COVID-19 positive homes had positive PCR tests [178]; in Germany 0.69% [179], and Northern Italy 3.8% of 920 and 277 household cats, respectively, tested positive for SARS-CoV-2 antibodies [180]. Interestingly, nine cats in very close contact with COVID-19 patients on a veterinary campus did not contract the virus [181]. While this last study had a small sample size, it is notable because the cats were in such close proximity with infected human patients and many of these patients reported sharing beds with and allowing face- and hand-licking from their cats.

In reported cases of felids that tested positive for the virus, the animals exhibited a range of clinical signs including ocular discharge, wheezing, coughing, sneezing, and vomiting [83,84,182]. Bronchiectasis, alveolar-interstitial syndrome, and virus-associated tissue damage were observed in infected felids in the Bronx Zoo along with respiratory and gastrointestinal symptoms [42]. In Switzerland, an infected cat displayed decreased appetite, sneezing, and coughing; the cat was diagnosed with an upper respiratory tract infection [39]. Data such as these obtained from cats in the home or in captive exhibition indicate that felids are susceptible to infection from other infected cats or from infected human caregivers.

Laboratory studies utilizing cats have also found them to be susceptible to SARS-CoV-2. Inoculated domestic cats in experiments by Shi et al. [77] exhibited respiratory symptoms similar to those seen in humans, as well as lesions in the mucosa epitheliums of the trachea and nasal passages, and lungs. Chiba et al. [183] also found lesions in the lungs of experimentally inoculated cats.

In an effort to determine possible transmission pathways, Bao et al. [184] intranasally inoculated six laboratory cats and then housed each with a naïve cat for 2 days beginning on day 1 post-inoculation. On day 3 post-inoculation, the formerly naïve cats were removed from their infected cagemates and placed in pair housing with another naïve cat. This process was repeated one more time, to provide researchers with cats who had been directly exposed to experimentally infected cats, as well as those who had been exposed to other exposed cats a total of two and three times removed from the initial infected animals. All cats were monitored for clinical symptoms, and blood samples, throat and rectal swabs were collected at nine different time points post-inoculation. At the end of 14 days post-inoculation or post-exposure, two cats from each group were necropsied to assess any resulting histopathology.

Inoculated cats showed elevated body temperatures, an arched back posture, and diarrhea, similar to symptoms seen in clinically diagnosed cats. Viral RNA collected in throat samples from these animals peaked on day 3 post-inoculation, and was undetectable by day 11. Necropsies revealed moderate interstitial pneumonia and damage to alveolar epithelial cells. Bronchiolar epithelial cells also showed damage. By comparison, cats exposed to the inoculated animals showed variable consequences. In cats exposed directly to the originally infected animals, viral RNA in throat swabs showed shorter shedding periods and attenuated peak levels than in those infected animals. As exposure “distance” increased from the originally infected cats, apparent presence of the virus as measured by the means described above decreased. The authors conclude that while domestic cats can become infected with SARS-CoV-2 through close contact and exhibit mild to moderate symptoms of COVID-19, subsequent passing on of the infection to naïve individuals seems unlikely. Other studies of experimentally inoculated cats also suggest that COVID-19 typically presents in cats with milder or no symptoms (compared to humans) [185,186,187]. Thus, while cats remain susceptible to the virus when in direct contact with an infected human or conspecific, they may be somewhat ineffective carriers.

Direct transmission is suspected to have occurred between lions (*Panthera leo krugeri*) housed together in the Bronx Zoo, NY, where three lions were infected with SARS-CoV-2 [42]. It is likely that one lion contracted the virus through contact with an infected keeper and then transmitted it to the other two animals. In a case in Switzerland, two cats living in a COVID-19-positive household contracted the virus; one was symptomatic and one was not [39]. The bedding and fur of both cats were PCR positive for the virus. This was the only case found in the literature that reported testing of viral transfer via bedding and fur, and it opens the possibility of transmission through shared bedding. These possible modes of transmission are relevant to households with multiple cats, zoos, animal shelters, vet offices, cat cafes, or any other environment where cats come into direct and indirect (e.g., shared bedding) contact with each other, and with humans.

Stray cat populations could also be at risk for the spread of infection. Evidence of the virus in serum samples collected from stray cat populations has been detected in Wuhan, China (11 of 102 cats sampled from shelters or pet hospitals; [188]), Zaragoza, Spain (4 of 114 stray cats [33]), and Lombardy, Italy (1 of 105 stray cats [189]), though at very low levels. Analysis of the Wuhan cohort suggests that these cats are contracting the virus from human contact, rather than transmitting it among themselves ([189]; for a review of anthropogenic infections in cats, see [40]). The potential viral infection in stray cat populations has significant welfare implications as it indicates a spillover from humans to free-ranging populations. Additionally, it raises the question as to whether wild or feral animal populations could serve as a viral reservoir. Transmission from other nonhuman animals to cats also appears to be possible as several cats that came into contact with infected mink on mink farms tested positive for SARS-CoV-2 antibodies [118].

There is evidence that previous infection with SARS-CoV-2 in domestic cats provides some protective immunity to reinfection. Chiba et al. [183] found that experimentally inoculated cats can transmit the virus to naïve cats, but attempts to reinfect cats were unsuccessful. Similarly, Bosco-Lauth et al. [186] found that previously exposed cats could not be reinfected. However, Gaudreault et al. [187] did find detectable levels of viral shedding after reinoculating cats. Once infected, the reinoculated cats were not, however, able to successfully transmit the virus to naïve cats. The authors conclude that while previously infected cats were able to become reinfected, the previous infection still did provide some immunity because the levels of viral shed resulting from the second infection were insufficient to transmit the virus.

Another concern particularly for domestic cats is the possible presence of virus with infectious capabilities in the feces of infected cats, since human cat caretakers commonly interact with cat feces through the cleaning of litterboxes. It is also possible for fecal virus to be transmitted to cat fur by their human caretakers, which might then be ingested when cats groom themselves. A number of studies have demonstrated persistence of the SARS-CoV-2 virus in fecal samples taken from infected humans, even after pharyngeal samples test negative (e.g., [190,191,192]). Orofecal transfer of the virus thus remains a realistic concern for both species.

Evidence of SARS-CoV-2 has been found in cat fecal samples (both in clinically reported cases [178] and in laboratory settings), as well as in the fecal samples of humans. The presence of ACE2 expression in the gastrointestinal mucosa of domestic cats supports the possibility of the gastrointestinal tract being a replication site for the virus [77,176]. All of the infected big cats at the Bronx Zoo as well as another tiger that was asymptomatic shed detectable SARS-CoV-2 RNA in their feces [42]. Isolation of the virus from the fecal samples suggests the possibility that infectious virus may also be present in feces. Human caregivers and pet owners may thus be able to transmit the virus to their cats through lax hygiene practices, or contract the virus from their cats in a similar way when handling soiled cat litter or cleaning cages. The data suggest that it is in the best welfare interests of both species for humans who interact with the Felidae to take greater precautions and manifest good hand hygiene when caring for their charges.

### 2.6. The Canidae

Domestic dogs (*Canis familiaris*) are common pets, with over 470 million reported worldwide in 2018 [169]. Under the social restrictions imposed by government lockdowns in response to the COVID-19 pandemic, demand for companion animals and especially dogs skyrocketed; anecdotal reports suggest that humans in lockdown increased the demand for puppies, outstripping supply and driving up prices [193], and shelters completely emptied of animals up for adoption were also reported [22,23,24]. The Animal Medical Center in New York City, NY (USA), which touts itself as the world’s largest non-profit animal hospital, reported a 25% increase in new puppy and kitten clients concomitant with the pandemic [194]. The increased demand for dogs as companion animals at this time put these animals at increased risk for possible infection by infected humans.

Since more people who obtained dogs were working from home or otherwise were unable to leave their homes during the pandemic, dog interactions with and exposure to humans have increased during COVID-19. Adolescents have reported spending more time with their dogs during the pandemic [195], as have pet owners in general [196]. One study in the United Kingdom that surveyed families with dogs found that during the pandemic, about 70% of dogs spent more time with adults, and more than 86% of dogs were around children more often [197]. Dog walking, while typically thought of as a positive way to interact with one’s dog and get physical exercise at the same time, may in this unprecedented time have been deleterious; at least one survey of over 2000 people in Spain found that walking a dog increased the risk of COVID-19 contagion by 78% [198]. Additionally, it was hypothesized early on at the start of the pandemic that dogs may have served as immediate hosts for SARS-CoV-2 [35]. Publications such as these helped to stimulate research into the susceptibility of dogs to the virus.

Canines are also used for biomedical research. The USDA [92] reported 64,707 canines were used for regulated research activities in 2017. Dogs can suffer from their own variant of a betacoronavirus (such as is SARS-CoV-2)—canine respiratory coronavirus (CRCoV)—which presents with symptoms similar to those reported for humans infected with SARS-CoV-2 [199]. These similarities have led some to suggest that dogs and CRCoV may make good models to improve our understanding of the betacoronaviruses and ultimately SARS-CoV-2 [200], and provide another rationale for why dogs have been among the species evaluated in the search for the best animal models for studying SARS-CoV-2.

At the time of this paper, limited research has been conducted regarding the polymorphisms of the TMPRSS2 receptor in canines. However, the TMPRSS2 protein is conserved between humans and dogs, and there is little variability between the two species in this protein [201]. More research has been conducted in regards to canine and other animal ACE2 receptor polymorphisms. The amino acid sequence of the ACE2 receptor in dogs is 81% identical to humans [74]. However, dogs only have one of the four of the key amino acid sequences for the ACE2 receptor that have been linked to increased SARS-CoV-2 susceptibility [70]. They also show a single mutation (H34Y) that is not found in humans or cats; this mutation appears to be the reason for reduced susceptibility to SARS-CoV-2 in canines compared to these other two species [202]. A more recent study suggests that dogs lack some genes underlying the inflammatory immune response that is normative in humans infected with COVID-19, and that a reduced immune response to the virus is among the reasons for a milder presentation of symptoms in dogs, many of which are suspected to be a consequence of excessive cytokine responses to the virus [203].

The studies mentioned above were essentially in silico or in vitro, but in vivo studies similarly suggest that canine susceptibility to SARS-CoV-2 is limited. Laboratory transmission studies suggest that virus-inoculated dogs show limited susceptibility to SARS-CoV-2, and that virus-naïve dogs do not become infected when exposed to experimentally infected dogs, even when contact is direct. Shi et al. [77] intranasally inoculated five young beagles with SARS-CoV-2, and then housed them in the same room with two naïve dogs. Oropharyngeal and rectal swabs were collected from all dogs every other day post-inoculation. Viral RNA was detected in the rectal swabs of two inoculated dogs on day 2 post-inoculation, and in one dog on day 6 post-inoculation. No infectious virus was detected in any of the dogs at any time during the course of the study. Only two inoculated dogs showed seroconvertion as assessed by ELISA assay; all other inoculated dogs and the naïve dogs were seronegative. In a similar study, three dogs were intranasally inoculated with SARS-CoV-2 [186]. Oropharyngeal swabs, nasal washes, and blood samples were obtained at regular intervals post-inoculation, and clinical observations made daily to document any potential disease symptoms. None of the dogs ever exhibited any symptoms, nor was any viral shedding detected, though neutralizing antibodies were detected by day 14 post-inoculation. To date, the available data suggest that dogs may not be effective research subjects for COVID-19.

Reduced susceptibility does not mean that dogs cannot become infected with SARS-CoV-2. In Hong Kong and the United States, anecdotes of domestic dogs who were infected with by their human caregivers SARS-CoV-2 have been reported [85,204]. Both dogs in Hong Kong, a Pomeranian and German Shepherd, remained asymptomatic during their quarantine period, while a Pug in North Carolina (USA) showed minimal symptoms, including a mild cough and reduced appetite. These dogs had been tested for the virus because of their direct contacts with infected owners. The USDA maintains an updated database of animal cases on their website; as of the time of this paper, a total of 78 dogs in the USA were reported on this site as having tested positive for exposure to SARS-CoV-2 (23 by diagnosed by reverse transcription PCR, and 55 by measuring virus neutralizing antibody counts) [157].

A larger study sampling 603 dogs in Italy showed only 3.3% of dogs demonstrating SARS-CoV-2 antibody titers (measured in several different ways), and none of the dogs sampled exhibited any signs of disease. The percentage of dogs showing virus-neutralizing antibody titers correlated positively with the number of human cases of COVID-19 in the districts in which the dogs lived [180]. Similar results were found in a sample of 35 dogs in France [205], with higher rates of seropositivity for SARS-CoV-2 in dogs living in families with a COVID-19 positive family member. In a longitudinal study of 59 dogs living with at least one human with COVID-19, only 1 of the 59 were positive for the virus (assessed by RT-PCR sequencing). Seven of the dogs showed viral antibodies, but no seroreversion was detected in any of the dogs and no clinical symptoms were observed [206]. Thus, while it is possible for dogs to contract COVID-19 from humans, human-to-dog transmission appears rare. It is possible that more dogs have been exposed to and infected by the virus than we are aware of, considering how minimal symptoms have been in dogs testing positive for the SARS-CoV-2 virus to date. At present, the evidence suggests canine risk for SARS-CoV-2 is primarily from living in proximity to infected humans, but that humans are not at risk for canine-to-human transmission of the virus [207]. The data reviewed here also suggest that domestic dogs are less than ideal potential subjects for SARS-CoV-2 and COVID-19 research.

Besides their putative use as research subjects or their risk for disease because of exposure to infected humans, domestic dog welfare has been affected by the COVID-19 pandemic in other ways. In many respects, dogs seemed to alleviate some of the stress of the pandemic on their human companions. Previous research suggests that dogs facilitate human social interactions [208], and during the pandemic, the majority of participants with dogs surveyed in a study in the USA reported that they socialized more often with others than did participants without dogs, presumably in part through walking the dog outside [209]. A similar study of Spanish dog owners showed that pet dogs (more so than other humans) served as social companions for their owners during pandemic lockdowns and that attachment to their pets among dog owners increased during this time [210].

Such increases in human-dog interactions were not always to the benefit of the dogs, however. Pet owners in several studies reported that their dogs were “clingy-er” as a consequence of the lockdown, and described other behaviors indicative of increased separation distress [196,211]. Others have reported an increase in dog aggression and bite cases [212], especially of children [213]. Data such as these suggest that just as lockdowns mandated in response to the pandemic have stressed humans, they have also stressed our nonhuman animal companions. Indeed, some veterinary behaviorists fear an increase in problem behaviors in pet dogs as a consequence of the pandemic [214].

*Canis familiaris* is certainly not the only canid species, nor the only canid that routinely comes into close contact with humans. Although at the time of this writing, nothing was found regarding SARS-CoV-2 in other canid species, raccoon dogs (*Nyctereutes procyonoides*), a canid species frequently farmed for their fur, were found to be susceptible to SARS-CoV-2, and could transmit the virus through direct contact with conspecifics [215]. In this study, nine raccoon dogs were intranasally inoculated with the virus and then housed with three naïve conspecifics 24 h post-inoculation. Nasal, oropharyngeal, and rectal swabs as well as blood samples were collected at staggered intervals up to a period of 28 days post-inoculation. Moreover, at staggered times, sample animals in the study were sacrificed and autopsied. Viral RNA detection assays revealed that six of the original nine inoculated animals became infected with SARS-CoV-2. Infectious virus was detected in nasal and oropharyngeal swabs starting as early as day 4 post-inoculation in some individuals and in nasal swabs as late as day 16 post-inoculation in others. Similar to domestic canines, minimal clinical signs were detected; however, some pathologies were noted in the caudal regions of the olfactory cavities. The apparent susceptibility of raccoon dogs to SARS-CoV-2 argues for precautions to be taken on the part of fur farm personnel to decrease possible exposure of their animal charges to the virus, just as with mink.

### 2.7. The Rodentia

Small rodents, such as hamsters (the Cricetinae), mice (*Mus* spp.), rats (*Rattus* spp.), gerbils (*Meriones* spp.), and guinea pigs (*Cavia porcellus*) are common house pets and, thus, may come into frequent contact with humans. Some of these species are also common pests, and may come into contact with humans and human-generated refuse through their invasion of human habitations and trash heaps. Small rodents are also common research species; an estimated 111.5 million rats and mice were used as research subjects in the United States between 2017 and 2018 [216].

A comparative study of gene sequences for the ACE2 receptor and the protease TMPRSS2 across select species of mammal, reptile, amphibian, and bird revealed similarities between these variables in humans and wild rodent species, but not in common laboratory species [78,217]. That said, transgenic mice expressing human ACE2 receptors are susceptible to SARS-CoV-2, suggesting that mouse TMPRSS2 proteases are capable of interacting with the virus’ spike protein [137].

With respect to ACE2 receptor similarities between humans and rodent species, both Norway rats (*Rattus norvegicus*) and wild type mice (*Mus musculus*) share only 13 of the 20 critical amino acid residues with humans [78], and appear not to be susceptible to human coronaviruses; the same is true for most common generic laboratory strains of mice [218]. In vitro studies using HeLa cell lines that express mouse ACE2 show that SARS-CoV-2 is unable to use this particular ACE2 receptor variant [1], as do some in silico studies (e.g., [136]).

Such findings have led researchers choosing to use rodent models in their SARS-CoV-2 investigations either to modify the variant of virus that they employ in their work [218], or to use mouse variants produced through selective breeding and gene manipulation (e.g., [219]). Studies with transgenic mice (*M. musculus*) expressing human ACE2 receptors, for example, have shown SARS-CoV-2 replication in lung tissue concomitant with interstitial pneumonia and weight loss in animals that were intranasally inoculated with the virus [220]; no such results were obtained in wild type mice subjected to the same procedures.

While commonly commercially available *Mus* species do not appear to be sensitive to SARS-CoV-2 without genetic manipulation, some rodent species in the Cricetidae family do appear to be susceptible to the virus. An in silico analysis of 42 mammalian ACE2 protein sequences showed that Campbell’s dwarf hamster (*Phodopus campbelli*), the 13-lined ground squirrel (*Ictodomys tridecemlineatus*), the Chinese hamster (*Cricetulus griseus*), and the golden Syrian hamster (*Mesocricetus auratus*) retained protein sequences in their ACE2 receptors that were permissive to binding by SARS-CoV-2 [78]. This latter species (*M. auratus*) shares with humans 22 amino acid sequences in the genes for its ACE2 receptor [221], and in vivo experiments demonstrate that golden hamsters can be readily infected with SARS-CoV-2. Sia et al. [222] intranasally inoculated hamsters and found detectable viral RNA in the lungs by day 2 post-inoculation. Evidence of damage to olfactory epithelium was observed, along with inflammatory infiltrates throughout the respiratory system. Naïve hamsters housed with infected individuals in the same cage readily became infected themselves, but the communicable period for infected animals did not appear to last much longer than 6 days post-inoculation. Naïve animals housed in soiled bedding taken from infected animals showed little evidence of exposure, suggesting that fomites are not an effective transfer mechanism of SARS-CoV-2 in this species.

The only symptom reported by Sia et al. [222] was weight loss, but others have reported inflammation of the lungs, increased respiratory rate, and pulmonary edema [89,223,224], as well as damage to the olfactory epithelium likely resulting in at least transient anosmia [225]. Weight loss is also consistently reported in infected animals. Overall *M. auratus* appears to be susceptible to SARS-CoV-2, but develops only mild symptoms at best, and modest lung pathology.

Chinese hamsters (*Cricetulus griseus*) have shown similar sensitivity to SARS-CoV-2 inoculation, responding with relatively similar symptoms and lung inflammation [226]. In a direct comparison of response to SARS-CoV-2 inoculation in three hamster species (Roborovski dwarf hamsters {*Phodopus roborovskii*}, Campbell’s dwarf hamster {*P. campbelli*}, and djungarian hamsters {*P. sungorus*}), Trimpert et al. [227] showed minor detectable symptoms of infection in *P. campbelli* and *P. sungorus*, but significantly greater changes in body temperature and overall activity levels in *P. roborovskii*. This latter species also showed other clinical symptoms such as shortness of breath and snuffling, and histological evidence of extreme lung damage. By day 3 post-inoculation, infected *P. roborovskii* showed compromised welfare significant enough to warrant humane euthanasia. This makes *P. roborovski* at present the only hamster species showing mortality in response to SARS-CoV-2 infection.

Other Cricetidae species studied to date for their susceptibilities to SARS-CoV-2 include bank voles (*Myodes glareolus*) and deer mice (*Peromyscus maniculatus*). Ulrich et al. [228] investigated the susceptibility of bank voles to SARS-CoV-2 because of the ubiquitous presence of this species throughout Europe and, thus, its potential to become a viral reservoir, should the virus become established in *M. glareolus*. Intranasally inoculated animals showed no clinical symptoms, nor did any naïve animals housed in contact with inoculated individuals. Oral and nasal swabs taken from inoculated animals did test positive for virus using qRT-PCR, and rectal swabs from a few infected animals similarly tested positive. No evidence of transmission to naïve co-housed conspecifics was detected.

Fagre et al. [229] intranasally inoculated deer mice (*P. maniculatus*) with SARS-CoV-2 to determine the susceptibility of this species to the virus. Similar to Ulrich et al.’s rationale for testing viral susceptibility of *M. glareolus* in Europe, Fagre et al. noted the ubiquitous nature of *P. maniculatus* in North America and thus its potential as a reservoir species. They also noted that the ACE2 receptor in deer mice shares 17 of the 20 amino acid residues considered critical for effective binding of SARS-CoV-2 to the receptor. Autopsied animals at various time intervals post-inoculation showed substantial pulmonary hemorrhage, and viral RNA was also detected in lung tissue. By day 14 post-inoculation, however, lungs appeared healthy again, though viral RNA was detectable as late as day 21 post-inoculation. Other pathologies were noted in the olfactory epithelium and gustatory buds. Virus was also detected in several areas of the brain. Cohoused naïve conspecifics tested positive for viral RNA as detected in oral (but not rectal) swabs by day 2 post-contact.

The literature reviewed here suggests that with a few possible exceptions (such as the hamsters and some transgenic mouse lines), rodent species typically used as subjects in laboratory research, such as is demanded by the current pandemic, are unlikely to be of much use as effective models for how SARS-CoV-2 presents in humans, though a few wild species may be worthy of further investigation.

### 2.8. Putative Source and Intermediate Host Species: Bats, Pangolin, and Palm Civets

Many of the first SARS-CoV-2 patients have been linked to the Huanan Wholesale Seafood Market in Wuhan, China, where wildlife such as bats (Order Chiroptera) and pangolins (Order Pholidota) are sold [230]. It has been speculated that the novel virus derived from a variant in bats and spread through some other intermediate host (see below) to humans through contact in the market [231]. This theory is based on an observed 96% genetic similarity between the bat coronavirus RaTG13 and SARS-CoV-2 [1]. Normative mutation rates in humans suggest that RaTG13 and SARS-CoV-2 shared a common ancestor between 25 and 65 years ago [232], which probably puts bats out of the running as the immediate source for the current virus, but makes it likely that the virus may have gotten its start in bats and then made the jump to humans via another intermediate host.

Bats are considered by many to be natural reservoirs for many viruses [233], including *Ebola* [234] and other SARS-like coronaviruses [235]. An apparent high tolerance for viruses in the Chiropterans that are lethal to many other species has been well-noted in the literature [236,237], and may be an indirect consequence of the evolution of flight in these species [238]. These authors argue that the metabolic demands of flight have intersected with immune system and gut microbiology evolution in the chiropterans in a way that makes these animals uniquely able to tolerate a diversity of viruses, and unfortunately for other species, to be able to spread those viruses through their feces and saliva.

Even if SARS-CoV-2 originally derived from bats, the virus likely made the jump to humans through another animal [239]. SARS-CoV-2 belongs to a particular variant of coronaviruses called the betacoronaviruses, and recent data suggest that little evolutionary change was required in the putative chiropteran coronaviruses to bring about the current version that has proven so lethal to humans [240]. Pangolins were first hypothesized as the intermediate host species through which such a change might have come about, because they were found to have a coronavirus variant with a receptor-binding domain nearly identical to SARS-CoV-2.

Pangolins are exclusive insectivores filling much the same ecological niche as an anteater. Their bodies—about the size of a small to medium-sized dog—are covered in keratinaceous scales. There are eight extant species of pangolin (four in Asia and four in Africa). In the Asian markets, the meat from all species is highly valued, and the scales are used in traditional medical practices. As laws put in place to control wildlife trafficking increase, so too has the value of pangolin products, adding to their desirability as an indicator of prestige in an area of the world with a burgeoning middle and upper class.

The net result is that pangolin are now commonly touted as the most heavily trafficked and endangered animal group in the world [241], and the Convention on International Trade in Endangered Species of Wild Fauna and Flora (CITES) called for an international ban on trade in all eight species in 2017 [242]. In China, however, trade in some pangolin products was still allowed prior to June 2020, and thus the possibility exists for pangolin to have served as the viral vector for SARS-CoV-2 to make the jump to humans.

A study of tissue samples collected from 18 Malayan pangolin (*Manis javanica*) that had been impounded as part of anti-smuggling operations in China in 2017–2018 reveal two different sub-lineages of SARS-CoV-2-related coronaviruses. Sequence similarities in the pangolin-derived viruses to the variant currently affecting humans range from 85.5% to 92.4%. Of special note with respect to the pangolin as a possible intermediate species in the evolution of SARS-CoV-2 is the finding that the receptor-binding domain sequences in the pangolin viruses share a 97.4% similarity in amino acid sequences with SARS-CoV-2 [243]. Further investigation suggests, however, that the genes underlying this receptor-binding domain differ too greatly to make it a likely evolutionary ancestor of the virus that has resulted in COVID-19 [244]. Lam et al. [243] also note that the pangolin-derived viruses lack a cleavage site in the viral spike protein that is present in SARS-CoV-2 and suspected to be one reason for the virus’ successful human infection rate. Controversy has also developed regarding the original animals from which tissue was sampled in these early studies, and whether or not they reflect true status as an intermediate host, or simply viral transmission from infected humans through the wildlife trafficking process [245].

As indicated above, pangolin are already a highly endangered group of animals. As data suggesting that they may be an intermediate host species for SARS-CoV-2 becomes commonly known, there are mixed thoughts on how such knowledge may affect their wellbeing. On the one hand, traffic in these animals may decline if demand declines as a consequence of human wishes to avoid possible infection. On the other hand, some conservationists have feared retributional killings of species associated with the current pandemic such as bats and pangolins [246].

Other putative intermediate host species include the masked palm civet (*Paguma larvata*), a medium-sized viverrid native to India and Southeast Asia [247], and one that is commonly trafficked for food in this region. This species harbors another coronavirus (SARS-CoV) that was responsible for a previous human epidemic in 2003–2004 [248]. Palm civets became suspect as a possible viral host of SARS-CoV when four patients were diagnosed with the virus in Guangdong, China, after the World Health Organization had declared that the epidemic was over. Three of these patients were found to have had direct or indirect contact with palm civets through working or eating at restaurants that served this species. Later tests of the animals at the restaurants showed that all of them were positive for SARS-CoV [249]. Genetic analyses of SARS-CoV sequenced from palm civets in markets and farms, however, as well as a lack of SARS-CoV virus detected in wild populations suggests that the infected animals in the restaurant might reflect an asymptomatic viral reservoir among farmed civets remaining from the original pandemic. The direction of transmission of the original SARS-CoV virus—from civet to human, or human to civet—remains unclear. The possibility of the current virus SARS-CoV-2 becoming established in an animal species that then serves as a reservoir, potentiating repeated outbreaks remains a great concern.

## 3. Discussion

In this paper, we review what is known regarding the susceptibility of mammalian species commonly in contact with humans to the virus responsible for the current COVID-19 pandemic, SARS-CoV-2. Our purpose is to focus primarily domestic species whose frequent contact with or proximity to humans makes them of special interest as potential viral reservoir species, and to consider the welfare impacts of the pandemic on these species. For comprehensive reviews of additional species (see [88,250,251,252,253] for some examples).

The data available using a combination of methods (in silico, in vitro, and in vivo) suggest that the greater the genetic and structural similarities there are between a particular nonhuman animal species’ ACE2 receptor and the human ACE2 receptor, the more likely that species will be susceptible to SARS-CoV-2, whose entry into the cell appears at present to be dependent upon this receptor. The essential similarities are those in the binding domain portion of the ACE2 receptor that is favored by SARS-CoV-2, and observed differences between in vivo findings of apparent susceptibilities to the virus and those predicted by in silico analyses may be due to small differences in gene sequences that affect this binding domain. Some of these differences exist as common polymorphisms shared across several species. Similarly, the ability of species-specific TMPRSS2 protease variants to facilitate viral entry may also explain differences between what has been predicted from large-scale genetic analyses across a number of genera and what has been observed in experiments with small numbers of live animals. Finally, the expression of both ACE2 receptors and TMPRSS2 differs not only between individuals, but also between species, and may thus be the reason for the variability in symptoms expressed by those infected with the virus. Nonetheless, the data reviewed here suggest that mustelids (such as ferrets and especially mink), rodents in the Cricetidae family (especially hamsters), the Felidae, and nonhuman primates (especially the apes and Old World monkey species) may be at particular risk, while dogs and ungulates remain unlikely to contract the virus or to transmit it.

This does not mean, however, that the pandemic has not had a substantial impact on the welfare of many animal species. Though not significant in number (considering the millions of human cases world-wide), the number of cases in which humans have transmitted SARS-CoV-2 to their pets, domesticated animals, and animals in captivity is not negligible [88], and “reverse” zoonotic transmission of human disease to other species is not as uncommon as one might think (for a review, see [254]). The financial and health impacts of the virus on human caregivers of nonhuman animals also results in a toll on animal welfare, as surrender of pets may become a financial necessity, and the number of healthy people required to care for livestock is reduced. Backlogs in food animal production systems have resulted in compromised welfare as animals experience greater crowding, longer times in transit, potential incompetencies in slaughter processes, and in some cases, mass euthanasia due to a lack of marketability [255]. Below we summarize some suggestions that have been made to try to reduce the impact of the pandemic on animal welfare.

### 3.1. Protecting the Welfare of Livestock and Food Animals during the Pandemic

As noted previously in this paper, the impact of COVID-19 on the livestock industry has been profound. In the United States, slaughter and meat processing facilities were hard-hit by the pandemic [57], and the resultant slow-down in livestock had profoundly detrimental effects on animal welfare [58,255]. In one survey of veterinarians servicing dairy farms in Pakistan, half reported that the farms they served were experiencing feed shortages because of the pandemic, and body scores of livestock across all 14 farms surveyed declined during this time. Seventy-one percent of farms were also unable to gain access to veterinary medical supplies, further compromising the welfare of their animals [256].

Grandin [255] suggests several policy and practice changes to the food animal industry aimed at reducing the adverse impact of this pandemic, and similar events on animal welfare. These include improving and refining procedures for mass euthanasia of animals when circumstances require it and reducing the size of centralized meat processing and packing plants in favor of smaller, more distributed facilities each of which would not cripple so much of the industry if compromised. Identifying alternative feed resources has also been recommended as a strategy to reduce the impact of pandemic disruptions to feed access, as has restricting access to animals and animal housing to essential personnel [257].

### 3.2. Protecting the Welfare Companion Animals

In a few instances, animals originally infected by humans have been able to infect other conspecifics, e.g., [40,45,125] or humans [45]. In the interest of protecting themselves as well as their pets, then, pet owners are urged to diligently engage in safe hygiene and husbandry practices (such as those recommended by the Centers for Disease Control and Prevention (CDC)). Pet owners diagnosed with COVID-19 should strive to have others care for their pets while they are ill and avoid physical contact with the pet; when this is not possible, infected pet owners are advised to wear a mask and use other PPE as appropriate to reduce or prevent contact [258]. Similarly, people are advised to avoid contact with stray or wild animals and their waste and body fluids, and to employ social distancing, keeping themselves and their pets away from other pets.

Other welfare concerns for pets as a consequence of the pandemic derive from the disruptions in routine that pets have suffered because of lockdown and quarantine policies that kept their caretakers at home. Some dog training facilities have reported an increase in clients with dog aggression behavior problems and biting as a consequence of COVID-19 restrictions [212]. Because human-directed canine aggression is the number one cause of canine surrenders [259,260] and the most common reason for longer stays [261] and euthanasia of animals surrendered to shelters [262], such observations are a particular concern for those interested in animal welfare impacts of the pandemic.

One study of Italian pet cat and dog quality of life as reflected in owner responses to several standardized questionnaires showed that as owners reported greater financial losses as a result of the pandemic, they also reported greater impairments to their pets’ quality of life [263]. A survey of 6004 dog owners in the United Kingdom showed that, as a consequence of government-mandated lockdowns, dogs spent less time alone, were walked less often, and had fewer interactions with humans who were not part of their households or with other dogs [197]. In other surveys, dog owners have reported that their dogs during lockdown have been more demanding of attention [209,210,211]. These behaviors foreshadow possible separation anxiety as pandemic restrictions relax and dog owners return to work outside of the home. Veterinarians and animal behaviorists are concerned that separation anxiety and other behavior problems may increase as pandemic restrictions relax and pet owners return to working outside the home [214,264]. It is recommended that veterinary facilities, animal shelters, and animal trainers increase outreach efforts to educate pet owners about how to recognize such behavior problems and how to prevent or ameliorate them (e.g., [265]).

### 3.3. Protecting the Welfare of Animals in Zoos and Aquariums

The welfare of exhibited animals, such as those in zoos and aquariums has been compromised by the current pandemic. Several species of captive big cats and a troop of zoo-housed gorillas have become infected with SARS-CoV-2 and made ill through interactions with infected keepers [5,42]. Reductions in staff resulting from personnel illness and lockdown restrictions also likely reduced keeper interactions with their charges, which reduces opportunities for training and enrichment.

The USDA (among others) has posted recommendations for zoos and other captive wildlife facilities, aimed at protecting animals from infection by SARS-CoV-2 [266]. These recommendations include changes to employment policies (such as eliminating penalties for sick leave), as well as the training of all employees on the proper use of protective personal equipment (PPE) and establishing standard procedures for disinfecting enclosures and feeding and handling materials. They also include recommendations for handling visitor interactions, such as requiring visitors to wear face masks, redesigning visitor setbacks and walkthrough exhibits to prevent visitors from being able to come within 6 feet of particularly susceptible species (e.g., NHPs, nondomestic cats, and mustelids), and suspending hands-on encounters between visitors and susceptible species.

Similarly, the CDC has written a series of guideline and recommendation documents relevant to those concerned about the impact that the pandemic may have or is having on nonhuman animals [267]. These include recommendations for veterinarians, people working with others who have tested positive for COVID-19 and who have pets in the home, pet stores, pet distributors, pet breeding facilities, and zoos, and are essentially similar to those recommended by the USDA as described above.

Zoo visitors have often been found to have adverse effects on zoo animal behavior (e.g., [268,269,270]), but not necessarily for all species [271]. In one study of a group of captive gorillas in a zoo in the USA, the total absence of zoo visitors as a consequence of the pandemic lockdown produced no substantive effect on animal behavior [272]. Similar results were found in observations of the behavior of several species at zoos in Ireland and the United Kingdom [65]. An Australian aquarium, on the other hand, reported that its fish were “depressed” without their usual human visitors, and increased diver swim times in the larger tanks in an effort to improve their animals; welfare [273]. Zoos and aquariums are advised to monitor their charges carefully for behavioral and physiological indicators of distress as restrictions are lifted and visitors begin to return to their facilities, to ensure that their animal charges do not suffer as a consequence of this change in their environments [65].

### 3.4. Protecting the Welfare of Wildlife

Several reports have been made suggesting exposure of SARS-CoV-2 to wild and feral mink [47,48,49], and feral cats have demonstrated seropositive reactions to the virus in Spain [189]. The possibility of a viral reservoir becoming established in wild or feral species that may have contact with other susceptible nonhuman animal species or with humans is a serious concern [274].

Wildlife conservation workers are advised to reduce or restrict visitor access to sanctuaries and preserves, and reduce or eliminate human–wildlife interactions as much as possible [275]. These authors also recommend the same application of precautions that have been advised elsewhere, including the reduction of human-animal interaction, stringent hand hygiene, and the proper use of PPE when interacting with wildlife, wildlife products, or surfaces that have interacted with the same. Such safety precautions will reduce the risk to humans of many zoonoses, but hopefully also reduce risk of humans unwittingly transmitting SARS-CoV-2 to animals.

The pandemic may provide a unique opportunity to reduce human appetite for exotic wildlife products [28,276], and some have suggested that wildlife conservation agencies capitalize carefully on the new public awareness of zoonoses in advertising campaigns aimed at reducing demand for wildlife products and increasing social distancing [277]. These authors also call for a strengthening of wildlife protection laws, and stricter monitoring of places and processes by which wildlife products, legally or otherwise, enter the marketplace.

### 3.5. Protecting the Welfare of Research Animals

As researchers and lab workers became ill with COVID-19, common laboratory species suffered from irregular care [54]. While many commonly used species reviewed here (especially rats and mice) do not appear to be susceptible to SARS-CoV-2, many other laboratory species do (e.g., hamsters, Old World nonhuman primates). Thus, it is recommended that staff working with these animals continue the diligent use of PPE, washing hands thoroughly and regularly after handling animals, feed, or bedding, and taking care to reduce opportunities for cross-transfer. Careful monitoring by personnel of themselves as well of their animal charges is also advised in order to detect any symptoms of ill health and thus enable timely isolation of those that might inadvertently spread the virus [266,267].

## 4. Conclusions

Animals are susceptible to a number of different, sometimes taxon-specific coronaviruses, and some species (see above) are apparently susceptible to SARS-CoV-2, the particular coronavirus responsible for the current pandemic. Infection with this virus itself poses a threat to animal wellbeing. But so too does the human response to COVID-19. In the race to find effective treatments and vaccines, animals face additional welfare challenges as researchers seek the best animal models for their work. At present, in silico and in vivo data suggest that the greater the genetic and molecular similarities are of a given animal species’ ACE2 receptors (particularly in the binding domain regions of those receptors preferred by SARS-CoV-2) to those of humans, the greater likelihood that species will be capable of becoming infected with the virus. Similarly, the greater the similarity in the structure of TMPRSS2 between an animal species and humans, the greater susceptibility to the virus. Finally, the more similar the expression and distribution of ACE2 receptors and TMPRSS2 is in an animal species to expression and distribution of the same protein and protease in humans, the more likely that animal will display symptoms of COVID-19 similar to those seen in humans. Thus, identifying putative species at risk through in silico analyses of available gene sequences and confirmation of susceptibilities in those suspected species through in vivo tests of inoculation and resulting infection will be critical in identifying appropriate species for research or those particularly at risk. Reviews such as this one, and another summarizing data amassed by the World Health Organization (WHO) [251], will be helpful in reducing the number of animals used in this research, refining the way nonhuman animal work is done, and replacing less susceptible nonhuman animal subjects with those more appropriate for such research. As our understanding increases regarding the mechanism by which SARS-CoV-2 enters the cell and produces the cascade of physiological events that lead to the disease state COVID-19, we should be able to reduce the impact of this virus not just on humans, but on nonhuman animals that come into close proximity to humans. Understanding which species are most likely to contract the virus and to display symptoms most similar to those seen in humans will reduce the unnecessary deaths of species in research that are really unfit as animal models. The same precautionary practices that we have been using to protect ourselves and one another will also hopefully be of similar help in reducing the likelihood of human-to-nonhuman animal transmission.

## Figures and Tables

**Table 1:** Comparison of genetic polymorphisms of the ACE2 receptor in twelve mammalian species: Human (*Homo sapiens*), Chinese rufous horseshoe bat (*Rhinolophus sinicus*), greater horseshoe bat (*Rhinolophus ferrumequinum*), domestic cat (*Felis catus*), domestic dog (*Canis familiaris*), domestic pig (*Sus scrofa*), domestic ferret (*Mustela furo*), rhesus macaque (*Macaca mulatta),* golden Syrian hamsters (*Mesocricetus auratus*), Sunda pangolin (*Manis javanica*), mink (*Neovison vison*), and mouse (*Mus musculus*). Of note, the polymorphisms K31, Y41, N90, and K353 correlate with increased susceptibility of the species to COVID-19 [70,71,72,73,74,75,76,77,78,79].

Species	ACE2 Genetic Polymorphisms
Human (*Homo sapiens*)	A291P, (D346-348), N90, Y41, K353, K31 [70]
Chinese rufous horseshoe bat (*Rhinolophus sinicus*)	K31, Y41H, N82, N90, K353 [70]
Greater horseshoe bat (*Rhinolophus ferrumequinum*)	K31D, Y41H, N82, N90, K353 [70]
Domestic cat (*Felis catus*)	T27, F28, D30, K31, H34, D38, Y41, Q42, M82, E329, K353, G354, D355 [70]
Domestic dog (*Canis familiaris*)	K353 [74]
Domestic pig (*Sus scrofa*)	TGF, BJ01 [76]
Domestic ferret (*Mustela furo*)	DPP4, BMP2, NFIA, AXIN2, DAAM1, ZNF608, ME1, MGLL, LGR4, ABHD6, and ACADM, K31, K353 [70,77]
Rhesus macaque (*Macaca mulatta*)	K31, E35, D38, M82, K353, N90, Y41 [70]
Golden Syrian hamster (*Mesocricetus auratus*)	K31, E35, D38, M82, K353 [70,78]
Sunda pangolin (*Manis javanica*)	K31, E35, D38, M82, K353 [70,78]
Mink (*Neovison vison*)	K353 [74]
Mouse (*Mus musculus*)	K31, M82, and K353 are present in genetically engineered mouse models as part of their human hACE2 [79]

**Table 2:** Comparison of the reported susceptibility, reported symptom presentation, and expected transmissibility in eleven mammalian species: human (*Homo sapiens*), Chinese rufous horseshoe bat (*Rhinolophus sinicus*), mink (*Neovison vison*), domestic dog (*Canis familiaris*), domestic pig (*Sus scrofa*), domestic ferret (*Mustela furo*), rhesus macaque (*Macaca mulatta),* golden Syrian hamsters (*Mesocricetus auratus*), Sunda pangolin (*Manis javanica*), domestic cat (*Felis catus*), and mouse (*Mus musculus*). Predicted transmissibility is based on reported susceptibility and reported symptoms.

Species	Reported Susceptibility	Reported Symptoms	Predicted Transmissibility to Humans
Human (*Homo sapiens*)	High [70]	Fever, cough, shortness of breath/difficulty breathing, fatigue, muscle body aches, loss of taste and/or smell [80]	High [81]
Chinese rufous horseshoe bat (*Rhinolophus sinicus*)	High [70]	No symptoms of pathology, transformation, of gut microbiome [82,83]	High
Mink (*Neovison vison*)	High [84]	Lung lesions, interstitial pneumonia [85]	High
Domestic dog (*Canis familiaris*)	Low [85]	No clinical symptoms [86]	Low
Domestic pig (*Sus scrofa*)	Low [85,87]	No clinical symptoms [85]	Low
Domestic ferret (*Mustela furo*)	High [87]	Severe lung inflammation, reduced activity, occasionally cough [87], elevated body temperature and loss of appetite [77]	Low
Rhesus macaque (*Macaca mulatta*)	High [70]	Weight loss with rapid respiration associated with moderate interstitial pneumonia and virus replication both in the upper and lower respiratory tract [88]	Low
Golden Syrian hamster (*Mesocricetus auratus*)	High [88,89]	Severe alveolar flooding, lung consolidation, increased respiratory rate, weight loss, resolving inflammation [88,89]	Low
Sunda pangolin (*Manis javanica*)	High [70,86]	Intestinal Pneumonia, severe congestion and infiltration of inflammatory cells in the liver, kidney, lymph nodes, minor hemorrhage in alveolar ducts, and epithelial surface of bladder [90,91]	Low
Domestic cat (*Felis catus*)	High [70]	Ocular discharge, wheezing, coughing, and sneezing, mild respiratory, and digestive complications [83,84]	Low
Mouse (*Mus musculus*)	Low [79]	No clinical symptoms [79]	Low

## Data Availability

This paper is a review of the literature. As such, the data presented here are in the form of summaries of published work relevant to this topic; the data used in the present manuscript are therefore available in their entirety in the form of the references listed at the end of the paper.
